# Assessing What Counts: Evaluating and Refining Geriatric Nursing Competency Assessments Through Item Analysis

**DOI:** 10.1093/geroni/igaf122.498

**Published:** 2025-12-31

**Authors:** Yosefa Birati, Allison P Squires, Mattia Gilmartin, Paule-Sarah Fraiman, Janna Sheps, Ilana Peterfreund, Anna Zisberg

**Affiliations:** The Cheryl Spencer Department of Nursing Faculty of Social Welfare and Health Science, Hanaton, HaZafon, Israel; New York University, New York, New York, United States; New York University Meyers College of Nursing, New York, New York, United States; University of Haifa, Haifa, HaZafon, Israel; Bnai Zion Medical Center, Haifa, Hefa, Israel; Bnai Zion Medical Center, Haifa, HaZafon, Israel; University of Haifa, Haifa, HaZafon, Israel

## Abstract

The Geriatric Institutional Assessment Profile survey, developed as part of the Nurses Improving Care for Healthsystem Elders Program (NICHE), plays a critical role in evaluating healthcare providers’ knowledge and competencies in geriatric-care. With the growing demand for geriatric services, equipping healthcare professionals with essential knowledge is vital to ensuring high-quality, person-centered care for older adults. Knowledge assessments are essential for evaluating competencies, yet their effectiveness and reliability are often underexplored. This study evaluates the psychometric properties of the Clinical Geriatric Knowledge (CGK) questionnaire and General Knowledge (GK) assessment, validating these tools beyond their original development setting, and testing their applicability and reliability in an international healthcare context. The analysis was conducted among 303 nurses across two hospitals. Participants were categorized into high-achievers and low-achievers based on performance. Using item analysis, we evaluated item difficulty (DIF-I), discrimination index (DI), distractor effectiveness (DE), and internal consistency reliability (KR-20). Results revealed that 60% of items had acceptable difficulty, and 65% of CGK items, and 60% of GK items demonstrated good/excellent discrimination. Distractor analysis showed that 25% of multiple-choice items effectively used all distractors, and 45% contained one non-functioning distractor. KR-20 scores were moderate (0.58 for GK, 0.52 for CGK), indicating acceptable reliability for assessments covering diverse content areas. These findings highlight the effectiveness of these tools in evaluating geriatric nursing competencies and revealing opportunities to optimize item design, particularly by balancing difficulty and refining distractors. This work contributes to advancing geriatric care and education, ultimately improving the quality of care for older adults.

